# Fully Integrated Photoacoustic NO_2_ Sensor for Sub-ppb Level Measurement

**DOI:** 10.3390/s20051270

**Published:** 2020-02-26

**Authors:** Yang Dong, Mingsi Gu, Gongdong Zhu, Tu Tan, Kun Liu, Xiaoming Gao

**Affiliations:** 1 Anhui Institute of Optics and Fine Mechanics, Chinese Academy of Sciences, Hefei 230031, China; dongyk@mail.ustc.edu.cn (Y.D.); mingsigu@mail.ustc.edu.cn (M.G.); gdzhu@aiofm.ac.cn (G.Z.); tantu@aiofm.ac.cn (T.T.); liukun@aiofm.ac.cn (K.L.); 2 Science Island Branch of Graduate School, University of Science and Technology of China, Hefei 230026, China

**Keywords:** nitrogen dioxide, photoacoustic spectroscopy, integrated sensor, detection limit

## Abstract

A fully integrated photoacoustic nitrogen dioxide (NO_2_) sensor is developed and demonstrated. In this sensor, an embedded photoacoustic cell was manufactured by using an up-to-date 3D printing technique. A blue laser diode was used as a light source for excitation of photoacoustic wave in the photoacoustic cell. The photoacoustic wave is detected by a sensitive microelectromechanical system (MEMS) microphone. Homemade circuits are integrated into the sensor for laser diode driving and signal processing. The sensor was calibrated by using a chemiluminescence NO–NO_2_–NO_X_ gas analyzer. And the performance of this sensor was evaluated. The linear relationship between photoacoustic signals and NO_2_ concentrations was verified in a range of below 202 ppb. The limit of detection was determined to 0.86 ppb with an integration time of 1 s. The corresponding normalized noise equivalent absorption was 2.0 × 10^−8^ cm^−1^∙W∙Hz^−1/2^. The stability and the optimal integration time were evaluated with an Allan deviation analysis, from which a detection limit of 0.25 ppb at the optimal integration time of 240 s was obtained. The sensor was used to measure outdoor air and the results agree with that obtained from the NO–NO_2_–NO_X_ gas analyzer. The low-cost and portable photoacoustic NO_2_ sensor has a potential application for atmospheric NO_2_ monitoring.

## 1. Introduction

As one of the main air pollutants, nitrogen dioxide (NO_2_) is mainly produced in engine combustion processes. High-concentration NO_2_ is toxic if inhaled, and a long-term exposure in low concentration can also cause airway inflammation and other respiratory effects. In addition to delivering direct effects, the existence of NO_2_ promotes the formation of acidic aerosols, which are strongly harmful to buildings and pedestrians, and also leads to the rising of the ozone level near the ground [1]. The typical NO_2_ mixing ratio in the atmosphere is a few tens of ppb, but may be one or more magnitude higher near its sources, such as rush-hour roads and airports [2,3]. The Ambient Air Quality Standards in P.R. China sets the safety limit to average 40 μg∙m^−3^ annually and average 200 μg∙m^−3^ hourly. The equivalent volume fraction is about 21 ppb and 106 ppb under 25 °C and standard ambient air pressure. To monitor this level of NO_2_ and locating the sources of pollution, a compact, sensitive and low-cost sensor is urgently needed to detect the concentration of NO_2_.

Methods to measure NO_2_ concentration have been widely studied. The techniques based on chemiluminescence are routinely used to determine NO and NO_2_ concentration in gas mixtures [4]. Chemical sensors with electrical conductance responses [5] or color responses [6] are possible to measure the concentration of NO_2_ from tens of ppb to hundreds of ppm. Spectroscopic methods, such as cavity ring-down spectroscopy [7,8], broadband cavity-enhanced absorption spectroscopy [9], laser-induced fluorescence method [10], and multi-wavelength quantum cascade laser spectroscopy [11], usually have detection limits of ppb or sub ppb levels, with sophisticated optical setup.

In recent years, trace gas detection using photoacoustic spectroscopy (PAS) receives wide attention and research. PAS is an application of photoacoustic (PA) effect, i.e., the phenomenon that the modulated or pulsed light illuminates materials to generate sound. This sound generation is caused by heat release from the excited molecules that absorb incident photons. The sound wave can be amplified acoustically by using a resonator and then transformed into electronic PA signals by a commercial microphone or other sound-sensitive detectors. Through subsequent signal processing procedures, an appropriate frequency component, usually at the same frequency as the resonance frequency of the resonator, can be obtained. The amplitude of this frequency component is proportional to the concentration of absorber under the circumstance of weak absorption.

Many researches on PAS were made to promote the performance of NO_2_ sensors. In 1996, R.L. Pastel and R.C. Sause reported a detection limit of 400 ppb by using a dye laser operating near 454 nm [12]. In 2001, V. Slezak reported a pulsed photoacoustic spectroscopy setup for measuring NO_2_ concentration in Nitrogen (N_2_) [13]. Two years later, V. Slezak et al. presented their NO_2_ trace detection using continuous and pulsed lasers at 532 nm, with a detection limit of 20 ppb and 15 ppb [14]. In the same year, G. Santiago et al. used a sound card in a personal computer to sample the PA signals and processed them on the PC, reaching a detection limit of 50 ppb. In 2006, M. Pushkarsky et al. reported a sub-ppb level detection of NO_2_ by using a room-temperature quantum cascade laser (QCL) [15]. H. Yi et al. introduced off-beam quartz-enhanced technique into PAS in 2011, obtaining the minimum detectable concentration of about 18 ppb [16]. In 2015, J. Peltola et al. reported their research on cantilever-enhanced PAS, with a detection limit of 50 ppt [17]. In 2017, X. Yin et al. [18] and T. Rück et al. [19] reported their work for ppt-level NO_2_ detection by using a differential PA cell and a PA cell with Brewster windows, respectively, both of which have a small size, but their commercial instruments to process the PA signals are expensive and bulky. In 2019, J. Kapp et al. reported their work on a ultraviolet light-emitting diode based photoacoustic sensor [20], which used their custom signal processing circuits, and the PA cell was carefully designed to reduce the background noises. The sensor has a noise equivalent concentration of 32 ppb, while the integration time is 1.14 s.

In this paper, we propose our work on building a fully integrated NO_2_ PA sensor. The structure of this PA sensor was all 3D printed, including a cylinder resonator, gas buffer chambers, laser case temperature controlling box, and other structural parts. A 3D printing technique has been well-developed over recent years. It is also known as a kind of additive manufacturing (AM) technique, because of its special process to synthesize objects. This unique synthesizing mechanism makes 3D printing technique suitable for manufacturing some dimensionally small but structurally complex objects. Since there are several kinds of structures assembled in the PA cell, it is much easier to build prototypes of the PA cell with 3D printers. As to the selection of light sources, the broad absorption spectrum of NO_2_ from 250 to 650 nm enables the application of a commercial blue laser diode in NO_2_ concentration measurement. This commercial high-power laser diode, driving circuits, home-made signal processing circuits and a communication port, were integrated into the sensor to minimize its size. The performance of this sensor was evaluated with experiments.

## 2. Sensor Design and Experimental Setup

### 2.1. Laser Source

**Figure 1:**
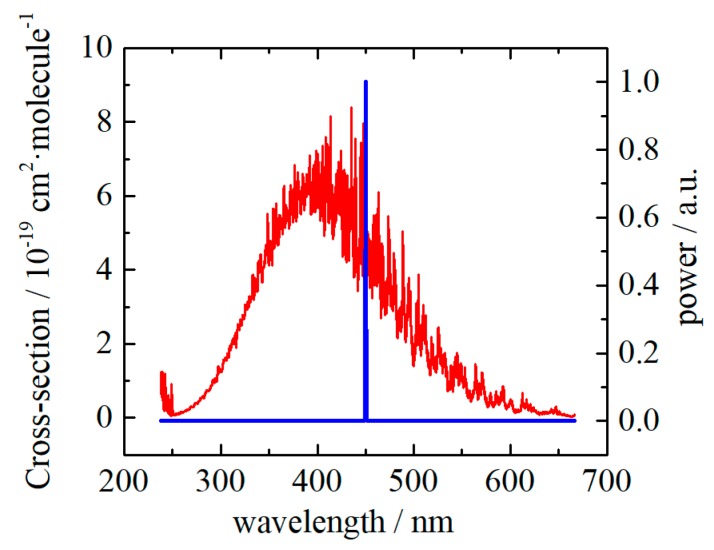
NO_2_ absorption cross-section in the range from 240 to 670 nm, compared with the laser emission spectrum. The laser has a peak emitting power at 450 nm when the case temperature is 25 °C, and the wavelength drift is about 0.067 nm∙°C^−1^.

The absorption cross-section of NO_2_ from 238 to 1000 nm has been measured with a Fourier transform spectrometer by A.C. Vandaele et al. [21]. The maximum absorption cross-section of 7.4 × 10^−19^ cm^2^∙molecule^−1^ is located at 414 nm. However, photochemical dissociation of NO_2_ occurs at the light wavelength of below 415 nm, which may induce a nonlinear PA effect when quantitatively detecting. Considering the cost and availability in the market, a blue laser diode (LD) whose emitting wavelength is 450 nm at 25 °C (PL TB450B, Osram, Munich, Germany) was selected. The emission spectrum of the LD, compared with the NO_2_ absorption cross-section, is shown in Figure 1. Since the wavelength drift of the LD is about 0.067 nm∙°C^−1^, a temperature controlling system, including a thermal electric cooler (TEC) (TEC1-031140, Pengnan Tech., Xiamen, China), a platinum resistor (M222, Heraeus, Hanau, Germany) and a home-made controlling circuit, was used to stabilize the case temperature of the LD at 25 °C. The maximum optical output power is 1.6 W, while the operating current is 1.5 A. A home-made current source with modulation input supplies square-wave current to the LD. The duty cycle of the square-wave current is 50%. A lens with anti-reflection coatings focuses the beam at the center of the resonator.

### 2.2. PA Cell

To keep the sensor compact, a PA cell that has the same cross-section with the temperature controlling box of the LD was designed. This PA cell has a dimension of 56 mm × 35 mm × 35 mm, made with 3D printing technique. The resonator inside the PA cell has a length of 30 mm and an inner diameter of 5 mm. In order to minimize flow noises, buffer chambers were designed at both ends of the resonator. The cylinder buffer chambers have a radius of 14 mm, and the length is 11 mm. Each buffer has a tube stick out from the wall as a gas inlet and outlet, and the inner diameters of the tubes are 1 mm. A microphone with a sensitivity of 0.22 V∙Pa^−1^ at 1 kHz (EK-23133-000, Knowles Electronics, Itasca, USA) is installed at the midpoint of the resonator. A small hole connects the resonator and the microphone for detecting the acoustic wave.

### 2.3. Signal Processing Circuits

Although the resonator amplifies sounds acoustically and the microphone has a fairly high sensitivity, the PA signal obtained is too weak. So some circuits are designed carefully to promote the signal to noise ratio (SNR). The power supply to the microphone is regulated and filtered with an inductor-capacitor filter. And the pre-amplification circuit has a gain of 250 by using a 4-stage amplifier. The microphone is directly soldered on the backside of the printed circuit board to avoid extern coupled noise.

**Figure 2:**
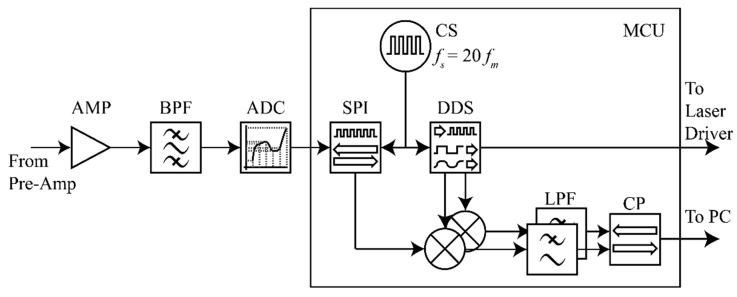
Digital lock-in amplifier to process the signal from pre-amplifier and offer modulation output to the laser driver. AMP: 4-stage amplifier; BPF: Band-pass filter; ADC: analog-to-digital converter; SPI: Serial Peripheral Interface; DDS: Direct digital synthesize; LPF: low-pass filter; and CP: calibration module and communication serial port.

Another part of the signal processing circuit is a microcontroller-based digital lock-in amplifier (DLIA), as shown in Figure 2. Recently, several kinds of DLIAs have been studied, which, however, either use expensive and hard-to-develop integrated chips like Digital Signal Processor (DSP) [22] or Field-Programmable Gate Array (FPGA) [23], or employ the microcontrollers (MCU) [24] for only low frequency signal processing, with low precisions acquired, due to the limited computing power and lack of floating processing ability. As the rapid development of integrated circuits, a new MCU with a floating processing unit and digital signal processing instructions now is available for the DLIA as the sampling controller and computing core. Once a signal enters the DLIA, a 4-stage band-pass filter will remove the out-of-band noises. Then the filtered PA signal is amplified to the level compatible with the reference of an analog-to-digital converter (ADC) by a 4-stage amplifier whose gain is set to 1000. Controlled by the MCU, the ADC samples and converts the signals at a sampling frequency of 20 times the modulation frequency.

At this sampling frequency, digitalized PA signals are processed by a software-based phase-sensitive detector (PSD) in the MCU. A direct digital synthesizer (DDS), which is also implemented by software, provides a pair of orthogonal reference sine waves to the PSD. Because the ratio of sampling frequency to modulation frequency is fixed, this implementation is as simple as building a look-up table storing a single cycle of phase biased sine waves. The demodulation parameters, such as the roll-off slope and the integration time, can be adjusted in the software. Considering the balance of processing power, signal-to-noise ratio and bandwidth of the sensor, these parameters are set to 12 dB/Oct and 1 s. A two-stage infinite impulse response (IIR) filter with a corresponding cut-off frequency of 125 mHz is implemented in the PSD. Theoretically, the output of DLIA is proportional to the absorption, and hence also proportional to the concentration of NO_2_, when the power of incident light is determined. The MCU can upload the signals onto a PC through a serial port.

### 2.4. Laboratory Setup

**Figure 3:**
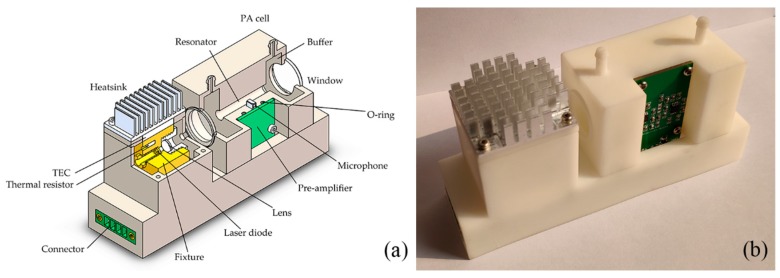
(**a**) A 3D model of the assembled sensor; (**b**) the entity. The dimension is 120 mm × 65 mm × 35 mm.

The dimension of the assembled NO_2_ sensor (shown in Figure 3) is 120 mm × 65 mm × 35 mm. To optimize the parameters and evaluate the performance, a laboratory setup is established for the sensor. An optical power meter is used to measure the power of exit beam, and it can be replaced by a beam dump (GCX-M02, Daheng Optics, Beijing, China) after the power is determined, to avoid potential harm caused by the high-power laser. By using a gas mixing system (N-4000, Environics Inc., Tolland, USA), NO_2_–N_2_ mixtures with different concentrations from pure N_2_ to the maximum 202 ppb are generated by diluting NO_2_ in N_2_. The gas flow fed into the sensor is controlled by a gas flow controller (D07-19B, Beijing Sevenstar Electronics Co., Beijing, China). After going through the sensor, the flow is fed into a chemiluminescence NO_X_ analyzer (Model 42i NO–NO_2_–NO_X_ Gas Analyzer, Thermo Fisher Scientific Inc., Waltham, USA) to precisely determine the NO_2_ concentration.

## 3. Results and Discussion

### 3.1. Resonance Characteristic

The modulation frequency needs to be determined first to optimize the acoustic signal amplitude. Since the cross-section of the resonator is orderly smaller than the target acoustic wavelength, only a one-dimensional acoustic field along the length of the resonator is generated [25]. The resonance frequency of longitudinal modes can be obtained by:

(1) $$f_{n}=\frac{nc}{2(l+\Delta l)}n=1,2,3,\ldots$$

where n is the number of longitudinal modes, c is the speed of sound, l is the length of resonator, and Δl is the so-called end correction, which can be approximately calculated by Δl=0.6r, in which r is the inner radius of the resonator [25]. The sound speed in an ideal gas is given by:

(2) $$c=\sqrt{\gamma RT}$$

where γ is the adiabatic gas constant, R is the ideal gas constant, M is the molecular mass of gas, and T is the absolute temperature. Theoretically, the odd longitudinal modes are much stronger than the even ones, and the first longitudinal mode is the strongest one. Thus, the modulation frequency is set to this value. Under the condition of ~25 °C (~298.15 K) and low concentration of NO_2_ in N_2_ in the cell, the resonance frequency of the first longitudinal mode, i.e., the modulation frequency $f_{M}=5.24\;\mathrm{kHz}$.

**Figure 4:**
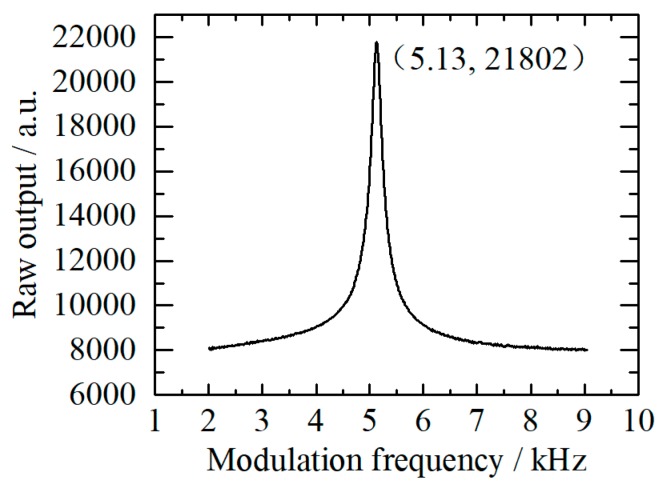
Amplitude-frequency response of the resonator.

Experiments were conducted to verify this theoretical resonance frequency. NO_2_–N_2_ mixture with a concentration of 202 ppb flows though the PA cell with a mass flow of 200 mL∙min^−1^ to keep the PA signal from any noteworthy flow noise. Modulation frequency was scanned from 2 to 9 kHz. The norm of orthogonal output was calculated in PC, so that the variation of the signal phase can be ignored. As shown in Figure 4, the resonance frequency of the resonator is 5.13 kHz, which was measured under the ambient pressure, ∼25 °C. This value is slightly smaller than the theoretical value. The deviation may be caused by the acoustic effect of buffers. The Q factor of the resonator is determined to 13.4 from the curve in Figure 4. The frequency is determined as the modulation frequency.

### 3.2. Performance Evaluation

**Figure 5:**
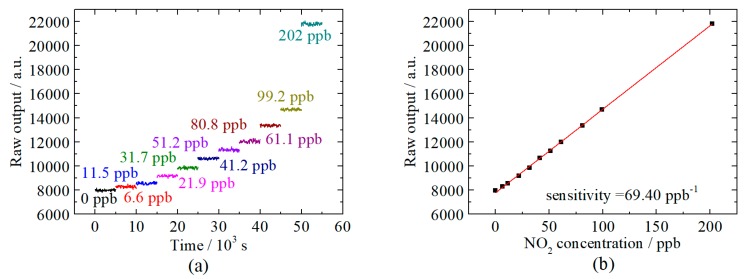
Calibration of the NO2 sensor. (a) The stable output of the sensor measuring the samples in a concentration range from 0 to 202 ppb. (b) The linear dependence of the output of the sensor upon the NO2 concentration.

The relationship between the raw output of the DLIA and the concentration of NO2 was measured in the range from pure N2 to 202 ppb. This range covers the safety limits and normal concentration in the polluted area mentioned before. The raw output is the digitalized voltages by the ADC. The stable output for NO2 with different concentrations is shown in Figure 5a.

Figure 5b shows a good linear relationship between the raw output and NO_2_ concentration, since the coefficient of determination is 99.944%. Due to the low standard deviation of the raw output, error bars are not illustrated in Figure 5b. Based on these results, the limit of detection (LoD) could be calculated by:

(3) $$\mathrm{LoD}=\frac{m}{\sigma }$$

where m is sensitivity, and σ is the standard deviation of the measured data. Here, m is the slope of the red fitting line in Figure 5b, which is 69.40 ppb^−1^. And σ can be retrieved from the measurement of pure N_2_, which is 59.97. So the LoD of this sensor could be determined to 0.86 ppb. The corresponding 1σ normalized noise equivalent absorption (NNEA) coefficient is defined as:

(4) $$\mathrm{NNEA}=\frac{\alpha_{\min}P_{0}}{\sqrt{\Delta f}}$$

where $\alpha_{\min}$ is the noise equivalent absorption, $P_{0}$ is the average laser power, and Δf is the bandwidth of the sensor. Thus, the NNEA of this sensor could be determined to 2.0 × 10^−8^ cm^−1^∙W∙Hz^−1/2^.

**Figure 6:**
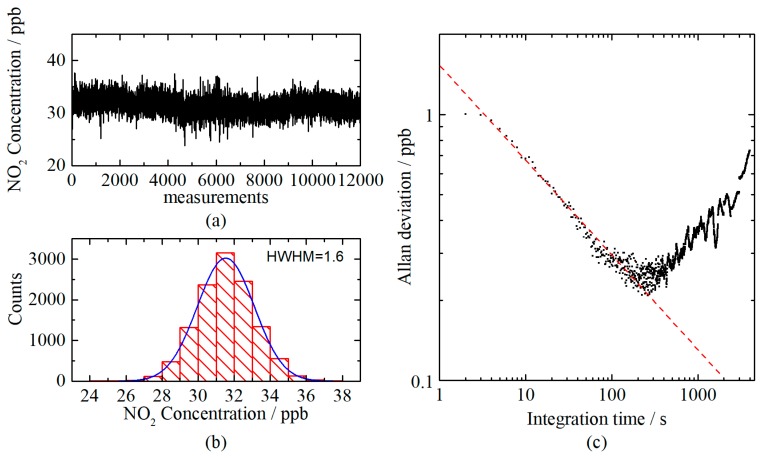
(**a**) Continuous measurements of 31.7 ppb NO_2_; (**b**) Histogram plot obtained from the measurements. The half width at half maximum (HWHM) of the Gaussian distribution curve is 1.6 ppb; and (**c**) Allan deviation plot of the measurements.

A long-running test was carried out for further evaluation. 31.7 ppb NO_2_ was measured 12,000 times continuously with an interval of 1 s. The measurement results are shown in Figure 6a. The Gaussian distribution of the measurement results, as shown in Figure 6b, has a half width at half maximum (HWHM) of 1.6 ppb. The stability and potential lowest detectable limit were evaluated with an Allan deviation analysis of the long-running test results, as illustrated in Figure 6c. The lowest detectable limit is found to be 0.25 ppb, with an integration time of 240 s.

**Figure 7:**
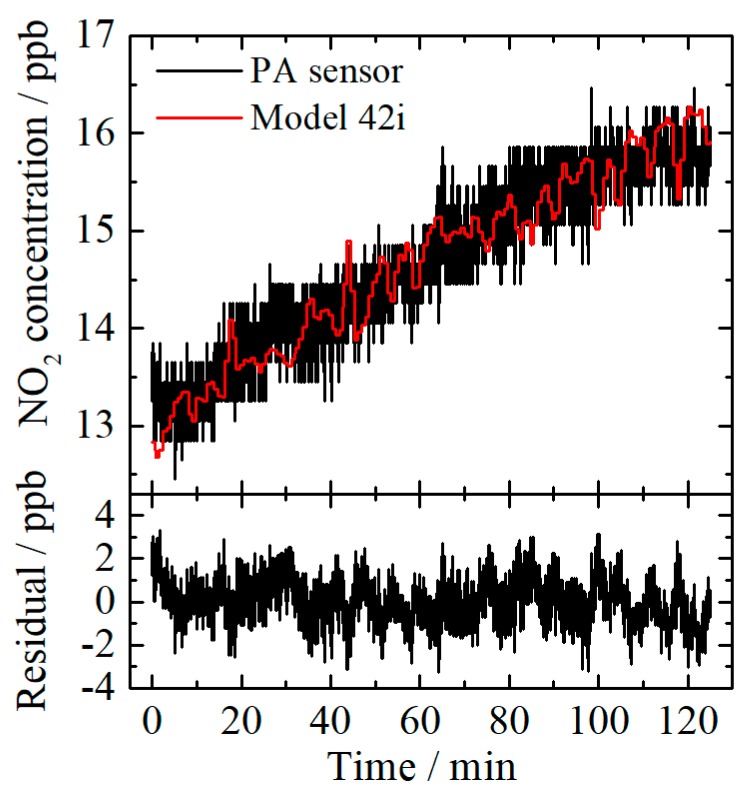
Comparison of outdoor NO_2_ concentration measured by using the 3D printed photoacoustic (PA) sensor and the Model 42i NO–NO_2_–NO_X_ Gas Analyzer. The standard deviation of the residual is 1.01 ppb.

For evaluating the practical performance of the sensor, outdoor air was filtered and fed into the experimental setup instead of the NO_2_–N_2_ mixture. The calibrated output of the sensor is shown in Figure 7, compared with the measurement results acquired from the Model 42i Gas Analyzer. The standard deviation of the residual is 1.01 ppb, which is slightly higher than the LoD obtained before. The consistent results imply that the developed NO_2_ sensor is capable for practical application, such as atmosphere NO_2_ monitoring.

## 4. Conclusions

A compact, low-cost, and sensitive photoacoustic sensor for NO_2_ trace gas sensing was developed. A 1.6 W blue LD, a 3D printed PA cell, and some homemade circuits for LD driving and signal processing, are assembled to form a fully functional sensor. With a small shape of 120 mm × 65 mm × 35 mm, the fully integrated sensor can measure NO_2_ concentration, getting power supply and communicating with other devices through a four-wire port. The LoD was evaluated to be 0.86 ppb with an integration time of 1 s. The corresponding NNEA is 2.0 × 10^−8^ cm^−1^∙W∙Hz^−1/2^. The high linearity in the range from pure N_2_ to 202 ppb was verified. The range covers the NO_2_ concentration in many polluted areas, such as roads and airports. A comparison between the sensor and a NO–NO_2_–NO_X_ gas analyzer in measuring outdoor NO_2_ concentration shows that the sensor has a practical potential. The performance evaluation indicates that the sensor can be employed to monitor environmental NO_2_ concentration or make exhaust analyses.
